# Pathological complete response to neoadjuvant cisplatin in BRCA1-positive breast cancer patients

**DOI:** 10.1186/1897-4287-13-S2-A8

**Published:** 2015-11-26

**Authors:** Tomasz Byrski, Tomasz Huzarski, Rebecca Dent, Elzbieta Marczyk, Marek Jasiowka, Jacek Gronwald, Jerzy Jakubowicz, Cezary Cybulski, Rafal Wisniowski, Dariusz Godlewski, Jan Lubinski, Steven A Narod

**Affiliations:** 1 Department of Oncology, Pomeranian Medical University. International Hereditary Cancer Center, Szczecin, Poland; 2 Department of Genetics and Pathology, Pomeranian Medical University. International Hereditary Cancer Center, Szczecin, Poland; 3 Sunnybrook Odette Cancer Center, Toronto, Ontario, Canada; 4 Oncology Institute, Krakow, Poland; 5 Regional Oncology Center, Bielsko-Biala, Poland; 6 Center for Epidemiology and Prevention, Poznan, Poland; 7 Women's College Research Institute, Toronto, Ontario, Canada

## Purpose

To estimate the frequency of pathologic complete response (pCR) after neoadjuvant treatment with cisplatin chemotherapy in women with breast cancer and who are BRCA1 mutation carriers.

## Patients and Methods

107 women with BRCA1 mutation and affected with breast cancer, who presented with stage I to III breast cancer between December 2006 and June 2014 received treatment with cisplatin 75 mg/m^2 ^every three weeks for four cycles, followed by mastectomy and conventional chemotherapy (AC). Information on clinical stage, grade, hormone receptor status and HER2 status was collected prior to treatment. Review of surgical samples was done in order to determine the pathologic complete response.

## Results

107 patients were included in the study. 93 patients were treated for first primary breast cancer and 14 patients had previously received treatment for a prior cancer. A pathologic complete response was observed in 65 of the 107 patients (61%).

## Conclusions

In a high proportion of patients with BRCA1-associated breast cancer platinum-based chemotherapy is an effective method of treatment.

